# Linking the Endocannabinoidome with Specific Metabolic Parameters in an Overweight and Insulin-Resistant Population: From Multivariate Exploratory Analysis to Univariate Analysis and Construction of Predictive Models

**DOI:** 10.3390/cells10010071

**Published:** 2021-01-05

**Authors:** Clara Depommier, Nicolas Flamand, Rudy Pelicaen, Dominique Maiter, Jean-Paul Thissen, Audrey Loumaye, Michel P. Hermans, Amandine Everard, Nathalie M. Delzenne, Vincenzo Di Marzo, Patrice D. Cani

**Affiliations:** 1 Metabolism and Nutrition Research Group, Louvain Drug Research Institute, Walloon Excellence in Life Sciences and Biotechnology (WELBIO), UCLouvain, Université Catholique de Louvain, Av. E. Mounier, 73 B1.73.11, 1200 Brussels, Belgium; clara.depommier@uclouvain.be (C.D.); rudy.pelicaen@uclouvain.be (R.P.); amandine.everard@uclouvain.be (A.E.); nathalie.delzenne@uclouvain.be (N.M.D.); 2 Quebec Heart and Lung Institute Research Centre, Université Laval, Quebec City, QC G1V 0A6, Canada; nicolas.flamand@criucpq.ulaval.ca (N.F.); vincenzo.dimarzo@criucpq.ulaval.ca (V.D.M.); 3 Pôle EDIN, Institut de Recherches Expérimentales et Cliniques, UCLouvain, Université Catholique de Louvain, 1200 Brussels, Belgium; dominique.maiter@uclouvain.be (D.M.); jeanpaul.thissen@uclouvain.be (J.-P.T.); audrey.loumaye@uclouvain.be (A.L.); michel.hermans@uclouvain.be (M.P.H.); 4 Division of Endocrinology and Nutrition, Cliniques Universitaires St-Luc, Avenue Hippocrate 10, 1200 Bruxelles, Belgium; 5 Centre NUTRISS, Institute of Nutrition and Functional Foods, Université Laval, Quebec City, QC G1V 0A6, Canada; 6 Endocannabinoid Research Group, Institute of Biomolecular Chemistry, Consiglio Nazionale delle Ricerche, 80078 Pozzuoli, Italy

**Keywords:** endocannabinoids, endocannabinoidome, human, metabolic syndrome, obesity, multivariate analysis, leptin, NEFA, LBP

## Abstract

The global obesity epidemic continues to rise worldwide. In this context, unraveling new interconnections between biological systems involved in obesity etiology is highly relevant. Dysregulation of the endocannabinoidome (eCBome) is associated with metabolic complications in obesity. This study aims at deciphering new associations between circulating endogenous bioactive lipids belonging to the eCBome and metabolic parameters in a population of overweight or obese individuals with metabolic syndrome. To this aim, we combined different multivariate exploratory analysis methods: canonical correlation analysis and principal component analysis, revealed associations between eCBome subsets, and metabolic parameters such as leptin, lipopolysaccharide-binding protein, and non-esterified fatty acids (NEFA). Subsequent construction of predictive regression models according to the linear combination of selected endocannabinoids demonstrates good prediction performance for NEFA. Descriptive approaches reveal the importance of specific circulating endocannabinoids and key related congeners to explain variance in the metabolic parameters in our cohort. Analysis of quartiles confirmed that these bioactive lipids were significantly higher in individuals characterized by important levels for aforementioned metabolic variables. In conclusion, by proposing a methodology for the exploration of large-scale data, our study offers additional evidence of the existence of an interplay between eCBome related-entities and metabolic parameters known to be altered in obesity.

## 1. Introduction

Obesity has reached pandemic proportions worldwide, mainly because of major lifestyle changes such as improper eating habits and lack of exercise. Nowadays, most therapeutic interventions aimed at preventing or reversing obesity remain limited in their impact. In this context, the need for further expanding our knowledge on obesity’s etiology is urgent. While studies on key single actors multiply and uncover new specific potential targets, these studies must imperatively be conceived in parallel with those covering the search for new key complex interplays between sets of biological subsystems. Among systems closely related to obesity-related comorbidities, the endocannabinoid (eCB) system has been shown to greatly influence host energy homeostasis and is involved in a number of physiological functions including food intake regulation, energy balance, lipid and glucose homeostasis, neuroprotection, inflammation, and pain modulation [1,2,3,4,5]. The eCB system includes a plethora of endogenous bioactive lipids, arachidonic acid (AA)-derived lipids, enzymes responsible for their synthesis and degradation, and membrane-bound receptors such as cannabinoid receptor 1 (CB_1_) and 2 (CB_2_) [6]. Bearing in mind that its complexity goes far beyond its simplified version, an expanded eCB system, or endocannabinoidome (eCBome), exists that encompasses, among others, two main bioactive lipid families, the *N*-acylethanolamines (NAEs) and the 2-acylglycerols (2-MAGs), which are congeners of the AA-derived and cannabinoid receptor activating endocannabinoids, *N*-arachidonoylethanolamine also known as anandamide (AEA), and 2-arachidonoylglycerol (2-AG), respectively. The first cluster includes *N*-palmitoylethanolamine (PEA), *N*-stearoylethanolamine (SEA), *N*-oleoylethanolamine (OEA), *N*-linoleylethanolamine (LEA), *N*-eicosapentanoylethanolamine (EPEA), and *N*-docosahexanoylethanolamine (DHEA), while the second cluster includes 2-oleoylglycerol (2-OG), and 2-linoleoylglycerol (2-LG), among others. In pathological conditions such as obesity, the levels of some of these mediators are altered in the blood and in several organs such as the liver, the brain or the intestine [7,8,9,10]. For example, levels of some NAEs were shown to be higher in individuals with elevated fat mass (FM), while increased levels of MAGs were found in individuals characterized by preferentially visceral fat distribution [11]. Obesity-related alterations in the eCB system are associated with excessive CB_1_ activation, which may favor increased food intake, mitigation of energy expenditure, and accumulation of fat in the adipose tissue [10,11,12]. On the other hand, eCBome mediators such as PEA, OEA, 2-OG, and 2-LG may influence energy metabolism by activating non-cannabinoid receptors such as peroxisome proliferator-activated receptor (PPAR) α, transient receptor potential cation channel subfamily V member 1 (TRPV1), G protein-coupled receptor 55 (GPR55) and 119 (GPR119) [13,14,15,16,17].

While an increasing number of studies began to shed light on the metabolism of these different mediators (for review see [2,18]), the complexity of the eCBome is not fully elucidated, nor its interconnections with other systems. To dismantle this puzzle, we considered data integration. Data integration reconciles, on one hand, the emergence of large-scale data sets, consecutive of improvement of high-performance analytical techniques and, on the other hand, the need to take into account the manner by which each biological system acts upon another. Several statistical approaches were proposed in the literature for the integration of two or more high-throughput data sets [19,20].

The present work aims at exploring the interplay between the circulating eCBome and metabolic parameters affected in the context of obesity. This was done first using an unsupervised approach to isolate relevant metabolic parameters and eCBome mediators; then regression models were constructed to predict a metabolic variable-response according to a specific eCBome subset. The circulating levels of 25 endogenous bioactive lipids belonging to the eCBome were quantified by LC-MS/MS in the plasma of overweight or obese individuals newly diagnosed with a metabolic syndrome and prediabetes.

Our preliminary explorative approaches displayed associations between subsets of eCBome mediators and the metabolic parameters leptin, non-esterified fatty acids (NEFA), lipopolysaccharide-binding protein (LBP), and fat mass (FM). A subsequent principal component analysis (PCA) performed on the eCBome confirmed the existence of a global link between the latter and those metabolic variables. Each variable response according to a specific eCBome subset was then modeled and validated internally. As such, we show that the predictive model constructed for NEFA response was performant enough to be applied to new observations. In contrast, our analysis performed on leptin and LBP failed to construct models for which the prediction error was acceptable when applied on new observations. However, when modeling was performed in a descriptive approach, our analyses suggested the importance of some specific mediators. Finally, analysis on metabolic parameter-related quartiles confirmed that certain eCBome mediators, considered as important in the regression model, were significantly higher in subjects whose levels of these variables were in the top quartiles.

While providing new insights into the complex interplay existing between metabolic parameters and the eCBome in the context of the metabolic syndrome, this paper also proposes an approach for methodological advances to better exploit information from large-scale data sets.

## 2. Materials and Methods

### 2.1. Subject Characteristics

The biological samples used in the present study originated from the Microbes4U cohort [21]. Individuals were recruited at the Cliniques Universitaires Saint-Luc in Brussels between 2015 and 2018, the study has been approved by the local ethical committee on July 2015 (comité d’éthique hospitalo-facultaire UCLouvain, Cliniques Universitaires Saint-Luc) under the number 2015/02JUL/369, and the study was registered at clinicaltrial.gov under the number NCT02637115.

The cohort consists of 32 overweight or obese subjects (body mass index (BMI) > 25 kg m^2^) newly diagnosed with a metabolic syndrome according to the National Cholesterol Education Program Adult Treatment Panel III definition, that is, at least three of the five following criteria: fasting glycemia > 100 mg dL^−1^; blood pressure ≥ 130/85 mmHg or antihypertensive treatment; fasting triglyceridemia ≥ 150 mg dL^−1^; high-density lipoprotein (HDL) cholesterol < 40 mg dL^−1^ for men, <50 mg dL^−1^ for women; and/or waist circumference > 102 cm for men, >88 cm for women. Subjects were also newly diagnosed with a pre-diabetic state as well as an insulin sensitivity <75% [22,23] (HOMA Calculator, University of Oxford, Oxford, UK). The subjects were naïve for medications influencing the parameters of interest (glucose-lowering drugs such as metformin, DPP4 inhibitors, GLP-1 receptor agonists, acarbose, sulfonylureas, glinides, thiazolidinediones, sodium-glucose cotransporter-2 inhibitors, insulin, lactulose, consumption of antibiotics in the previous 2 months before the inclusion, glucocorticoids, immunosuppressive agents, statins, fibrates, orlistat, cholestyramine, or ezetimibe). Anthropometric measurements were assessed including body weight (kg) and BMI (kg m^−2^). Waist and hip circumferences (cm) were measured using a flexible tape. Fat mass (kg) was assessed using electric bioimpedance analysis (Body Composition Analyzer, type BC-418 MA; TANITA).

### 2.2. Biochemical Analysis

Plasma samples were collected after an overnight fasting (8 h minimum). Different tubes were used: sodium fluoride-coated tubes for Milliplex analysis; and lithium-heparin-coated tubes for others analyses. One set of tubes was sent directly to the hospital laboratory for several blood analyses: including HbA1c (%); total cholesterol and HDL cholesterol. The other tubes were brought to the research laboratory and kept on ice. Plasma was immediately isolated from whole blood by centrifugation at 4200× *g* for 10 min at 4 °C and stored at −80 °C for further analyses.

Plasma NEFA were measured using kits coupling an enzymatic reaction with spectrophotometric detection of the reaction end products (Diasys Diagnostic and Systems, Holzheim, Germany) according to the manufacturer’s instructions.

Plasma LBP levels were measured using an appropriate ELISA kit (Human LBP ELISA kit, Hycult Biotech, Uden, The Netherlands, catalog no. HK315) according to the manufacturer’s instructions.

Plasma leptin levels were assessed in each blood sample in duplicate using a MILLIPLEX MAP Human Metabolic Hormone Magnetic Bead Panel and measured using Luminex technology (BioPlex; Bio-Rad Laboratories, Hercules, CA, USA) according to the manufacturer’s instructions.

The extraction of MAGs and NAEs in plasma was done as described previously [24]. In brief, samples were mixed with TRIS (pH 7.4, 50 mM) to a final volume of 500 µL. Toluene (2 mL) containing the ISTD was next added to the samples, which were vortexed for 1 min and centrifuged at 4000× *g* for 5 min without brakes. Samples were then put in an ethanol-dry-ice bath (−80 °C) to freeze the aqueous phase (bottom). The organic phase (top) was then collected and evaporated to dryness under a stream of nitrogen. Samples were reconstituted in 25 μL of HPLC solvent A (water with 0.05% acetic acid and 1 mM NH^4+^) and 25 μL of solvent B (acetonitrile/water, 95/5, v/v, with 0.05% acetic acid and 1 mM NH^4+^). A 40 μL aliquot was injected onto an RP-HPLC column (Kinetex C8, 150 mm × 2.1 mm, 2.6 μm, Phenomenex). Quantification was achieved using a Shimadzu 8050 triple quadrupole mass spectrometer using the same LC program as described previously [25]. For the MAGs containing unsaturated fatty acids, the data are presented as 2-MAGs although it represents the combination of 1(3)- and 2-isomers given the recognized acyl migration from the sn-2- to the sn-1 or sn-3 position.

Abbreviations used for the eCB system mediators and metabolic parameters present in this paper, are presented in Table 1. Between parentheses are abbreviations that were used in graphical outputs

### 2.3. Explorative Multivariate Approaches

Regularized Canonical Correlation Analysis (rCCA), implemented in MixOmics package (version 6.10.9, Bioconductor 3.12, University of Melbourne, Australia, University of Queensland, Brisbane and Université Paul Sabatier, Toulouse, France) [26] was used for a preliminary exploratory process to unravel potential links between two sets of data (i.e., metabolic parameters and eCB-related mediators). CCA allows to explore correlations between two sets of quantitative variables observed on the same subjects. More specifically, the statistical method maximizes the correlation between a linear combination of variables of the first set (metabolic parameters) and a linear combination of variables of the second set (eCB) [27]. An additional regularization step is included to deal with the high dimensionality problem [28]. rCCA was carried-out using the shrinkage method to estimate penalization parameters and *r*-coefficients were calculated between the first and second components. The rCCA-network is produced by calculating a pair-wise similarity matrix obtained from the latent components. Specifically, the sum of the correlations between the variables and each canonical variate was calculated. This allows to obtain a similarity value between each pair of variables [29]. To facilitate clarity, and to select the relevant variables for further analysis, cut-off values of 0.5 and of 0.55 were used for the circle plot and the network plot, respectively. A Spearman’s correlation matrix was then built using the variable revealed by the rCCA circle plot. To control type I error rate, the Holm’s adjustment was used. Following standardization, principal components analysis (PCA) on the measured eCB-related mediators was performed using the “factoextra” and “factoMinerR” packages (version 1.0.7 and 2.3, respectively). The color of the sample dots in the individual plot was set according to the concentration of the metabolic variable of interest. Linear regressions were then built between each metabolic variable of interest and the two first principal components summarizing the highest variance in the eCBome using the formula lm(variable~PC), the corresponding plots were constructed using the R package “ggplot2” (version 3.3.2). All statistical analyses were performed on RStudio (R version 3.6.3, Rstudio Team, Boston, MA, USA).

### 2.4. Variable-Response Modeling and Construction of Predictive Model

Regressions models were constructed on 100% of the observation (*n* = 32) to quantify NEFA, leptin, and LBP response according to a linear combination of the eCBs revealed by the rCCA. All models were built using either principal component regression (PCR) or partial least square regression (PLSR). Although these methods are slightly different, PCR and PLSR are both dimension reduction methods used to model a response variable when there are numerous potent predictors variables. In those regression models, the metabolic parameter was defined as the response variable and the subset of eCBs as the predictors. The importance of each predictor in the non-cross-validated quantitative model was assessed through estimation of the loading weight (lw), the significance multivariate correlation (smc), the selectivity ratio (sr), and variable importance in projections (vip). For each variable-response modeling, we compared the performance of PCR and PLSR and the final analytical method was chosen accordingly. Model performance was evaluated using the coefficient of determination (*R*^2^), the adjusted *R*^2^ which corrects the *R*^2^ for the number of observations and the number of predictors, and the root-mean-square error of prediction (RMSEP). For each model, the optimal number of components was assessed to capture the maximal variance for both the predictors and the variable to predict with a minimal number of components.

For internal validation of our models in a predictive approach, the dataset (*n* = 32) was randomly divided into a train set (80% of the full data set; *n* = 26) and a validation/test set (20% of the remaining observations; *n* = 6) using the “Caret” package in RStudio (Rstudio Team, Boston, USA) (version 6.0-86). The model was constructed on the train data and the model performance was thereafter verified using the test set. Data were normalized using standardization procedure. Models were constructed using the “pls” package (version 6.7-3). Leave-one-out cross-validation method was used to select the optimal number of PLS and PCR components and for internal validation of the models. For the cross-validated model, we performed approximate *t*-tests of regression coefficients based on jackknife variance estimates (jack test) to assess the statistical significance of the eCB predictors.

### 2.5. Univariate Analysis

For univariate analysis, selected metabolic parameters were categorized using quartiles. This creates three new data frames, with a supplemental column, in which the adherence of an individual to one of the quartiles was specified. For each metabolic parameter, a difference in the distributions of the concentrations of relevant eCBs, selected on the basis of the previous multivariate analysis, was assessed and visualized by boxplots. Kruskal–Wallis analysis or ANOVA was used to compare the eCB concentrations between the new categorical groups. The aforementioned statistical tests were chosen in accordance with normality tests. All statistical analyses were performed in RStudio (R version 3.6.3).

## 3. Results

### 3.1. Unsupervised Exploratory Approaches to Unravel an Association between the eCB System and Certain Metabolic Parameters

**Figure 1:**
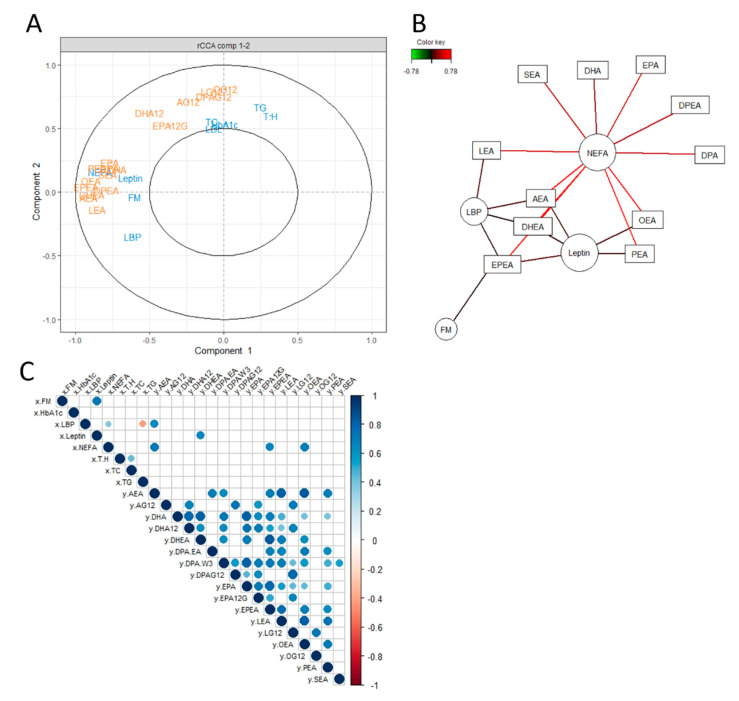
Unsupervised exploration of the relationship between measured eCB system-related mediators and biological parameters using regularized canonical correlation (rCCA) and correlation matrix. (**A**) Correlation circle plot allocating biological parameters and eCBome features along the main components derived from the integration of both data sets. The components correspond to the equiangular vector between *x*- and *y*-variates. The features in the area outside the inner concentric circle (radius < 0.5) were retained as significant and shown in the scatter plot. (**B**) Relevance network of top correlations between biological parameters (circles) and eCBome lipids (rectangles) with a cut-off = 0.55. Lines are colored according to the strength of the association score between two variables with red showing positive correlations. (**C**) Correlation matrix (Spearman with Holm’s adjustment); positive correlations are displayed in blue and negative correlations in red color. The color intensity and the size of the circle are proportional to the correlation coefficients. “X” refers to the first data set, the metabolic biological parameters while “Y” refers to the second data set, the endocannabinoids. Abbreviations: see Table 1.

To explore the interplay between mediators belonging to the circulating eCBome and biological parameters altered in the context of metabolic syndrome, we started our analysis by performing a CCA. CCA is a multivariate analytical method that enables the integration of two data sets in an unsupervised framework [20]. By maximizing the correlation between two respective linear combinations of the variables (called canonical variates), CCA can provide additional insights that could not be obtained by analyzing each pair of data alone. The metabolic variables included in the first data set cover glycemic, lipid, inflammatory, cardiometabolic, and anthropometric parameters. The second data set consists of the 25 eCB system-related mediators measured in the plasma. Given that the inclusion of metabolic variable in the analysis, in addition to the eCB, resulted in having more variables than observations, we regularized our CCA [28]. The graphical outputs of rCCA include a correlation circle plot (Figure 1A) and a relevance network (Figure 1B). In the circle plot, the variables are represented as vectors and the plot only shows the variables that correlate the most with each canonical variate. The nature of the correlation between two variables is visualized through the angles between two vectors. Accordingly, variables that correlated positively are projected on the circle plot close to each other, while variables that correlated negatively are projected in opposite direction [30]. The network displays graphically the strongest relationship between metabolic variables and eCBome mediators [30]. Both the circle and the networks generated with this exploratory analysis revealed the existence of a potential link between a specific subset of eCBome related mediators and the biological parameters leptin, NEFA, LBP, and FM (Figure 1A,B). Importantly, both graphical outputs are useful to help selecting the relevant variable (i.e., variables that correlated the most with the canonical variates). While the correlation matrix constructed on all the variables from our original data set would have been difficult to read, legibility was achieved by including only the variables revealed by the rCCA. Analysis of the Spearman’s rank correlation matrix confirmed the existence of a link between certain eCB-related mediators and leptin, LBP, and NEFA. In detail, the matrix showed that leptin was positively correlated to DHEA; NEFA was positively correlated to AEA, EPEA, and OEA, while LBP was positively correlated to AEA. Note that, probably as a result of the statistical constraints imposed and necessary for the veracity of the correlation matrix, no correlation was found regarding fat mass. This highlights the added value of combining different multivariate analysis as a first approach to explore the relationship between two data sets. The rCCA highlights more potential predictors than the correlation matrix. Finally, both approaches demonstrate strong collinear relationships between lipids belonging to the eCBome (Figure 1A,C), as it could have been predicted considering their metabolic relationships. In the following sections, we decided to focus our analysis on the links between the eCBome and the four metabolic variables, LBP, leptin, FM, and NEFA.

### 3.2. Linear Regression with Principal Components Analysis

To further explore the correlation existing between the selected metabolic features and the eCBome in our cohort, we performed a PCA on the whole quantified eCB data set. Like rCCA, PCA is an unsupervised multivariate dimension reduction technique. PCA constructs linear combinations of the different variables to identify the largest sources of variance in the data. It is then possible to realize two respective linear regressions between the two first principal components, accounting for most of the variance in the eCBome, and the metabolic parameter of interest. In other words, the variable to explain is the metabolic parameter and the predictor is one of the first two components obtained from the PCA.

**Figure 2:**
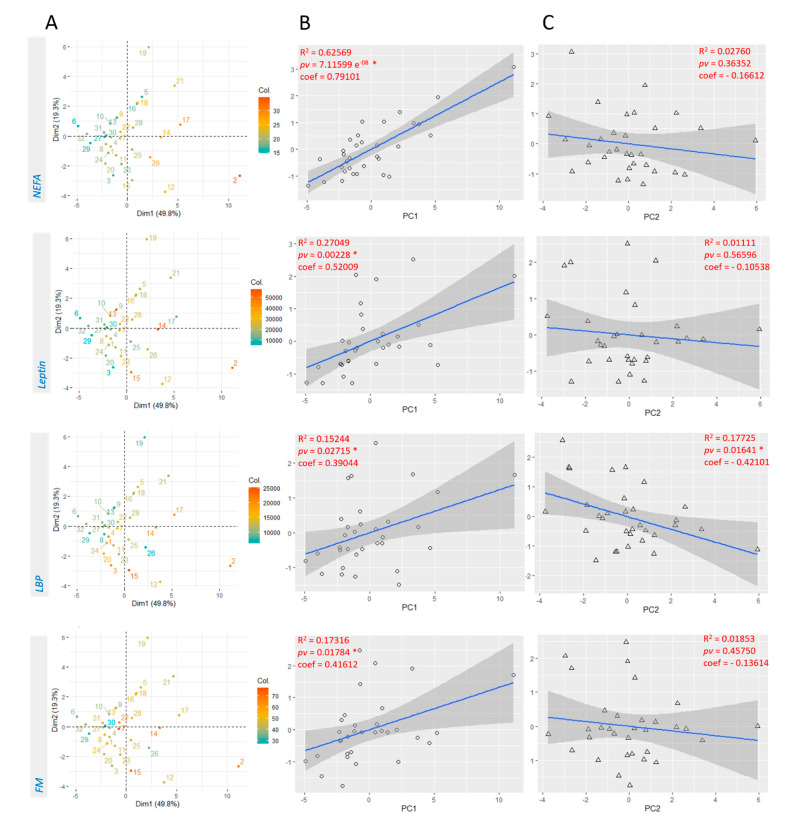
Deeper exploration of the relationship between measured eCB-related mediators and the biological parameters selected from rCCA using PCA. (**A**) Individual plots, the color gradient for each individual was established according to the concentration of the referred biological variable. (**B**) Scatterplots showing the linear relation between first principal components and the referred variable. (**C**) Scatterplots showing the linear relation between second principal components and the metabolic variable. The confidence interval is displayed in grey around the mean. (**B**,**C**) Each linear regression plot is accompanied by the R square, the *p* value and the coefficient of correlation (Pearson). Abbreviations: see Table 1.

This method is equivalent to the PCR which enables to cope with the multicollinearity issue at play, since the predictors in the linear regression were highly correlated with each other in the original data set (Figure 1C). The first principal component accounts for 49.8% of the overall variability in the quantified eCBome, while the second principal component explains 19.3% of it. The PCA generates an individual plot displaying the variability across individuals (Figure 2A). The four graphics only differ by the color of the individual dots. Indeed, dots color intensity was established to be the direct representation of the metabolic variable concentration measured in each participant. We observed that, for NEFAs and leptin, the color gradient follows the dimension 1 of the PCA. This suggests that individuals with a higher amount of leptin/NEFA are also characterized by a higher tone of eCBome mediators. This hypothesis was confirmed by linear regressions performed between each metabolic parameter and the first principal component that summarizes best the variance in the whole quantified eCBome (Figure 2B). More specifically, there are linear, positive, and significant relationships between NEFA, leptin, LBP, FM, and the first principal component (NEFA: *R*^2^ = 0.63, *pv* = 7.12 × 10^−8^; leptin: *R*^2^ = 0.27, *pv* = 0.0023; LBP: *R*^2^ = 0.15, *pv* = 0.027; FM: *R*^2^ = 0.17, *pv* = 0.018) (Figure 2B). Furthermore, there is a linear, negative, and significant relationship between LBP and the second principal component (*R*^2^ = 0.17, *pv* = 0.016) (Figure 2C). In the view of those results and given that no correlation was found for FM in the correlation matrix and few in the network, we next decided to focus our analysis on the three metabolic variables, LBP, leptin, and NEFA.

### 3.3. Modeling Variable Response According to Selected eCBs and Construction of Predictive Models

Starting from the mathematical principle that it is never in favor of the model to introduce too many predictors because this increases the risk of taking into account variables that do not help in the construction of the model but rather brings the reverse noise while reducing the adjusted *R*^2^, we seek to reduce the number of predictors. As a matter of fact, our preliminary exploratory analysis revealed—for each of the selected metabolic parameters—a close association with a specific subset of eCBome-related mediators. We then wondered whether a specific linear combination of this subset of eCBs and eCB-like mediators could predict variable response for leptin, NEFA, and LBP within our cohort in a descriptive approach. We then reconstructed the model with cross-validation and tested whether the model was efficient enough to predict variable response in new observations, in a predictive approach. Finally, for each of those models, we isolated the eCBome mediators that most influenced the model. For this step, we tested two different dimension reduction methods amenable with regression, the PCR and PLSR. Both methods are efficient to overcome multicollinearity issues because the new regressors variable become orthogonal to one another. For each variable, the model resulting in the best predictive and descriptive performance will be presented. Importantly, the subsequent search for relevant predictors in our models highlighted variables that were not previously revealed by the Spearman’s correlation matrix.

#### 3.3.1. NEFA Response Modeling

The best performance was obtained with PLSR, for which the first four components explained 91% of the variance in the eCBome while explaining 91% of NEFA variance. The non-cross-validated model had an adjusted *R*^2^ of 0.86 and an RMSEP of 0.30.

**Figure 3:**
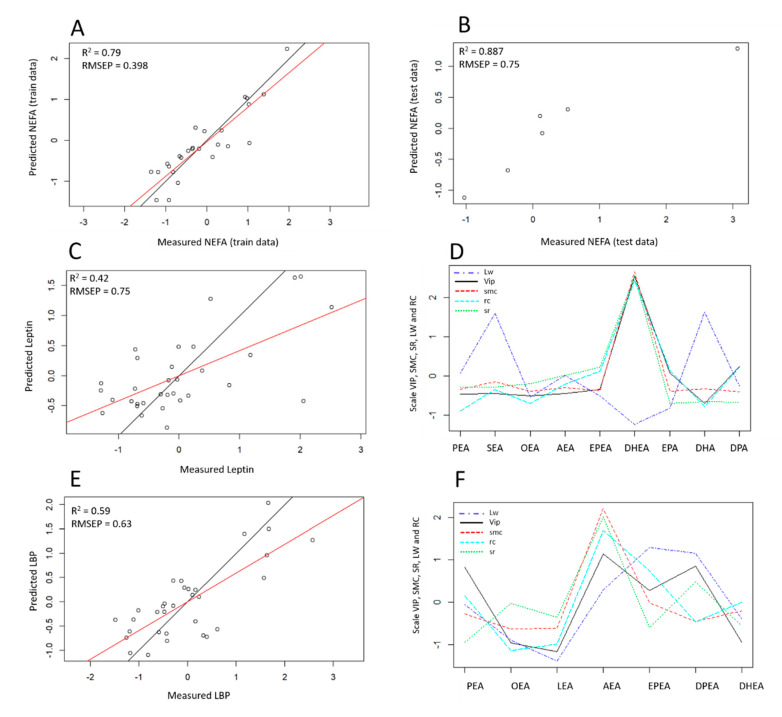
Modeling and validation of variable response according to a selected subset of the eCBome. (**A**,**B**) PLSR correlation plot between predicted and measured NEFA levels according to the selected eCBome mediator subset, illustrating the predictive quality of NEFA in the (**A**) training group (*n* = 26) and (**B**) the test group (*n* = 6). (**C**) PLSR correlation plot between predicted and measured leptin according to the selected eCBome mediator subset, constructed on all observations without cross-validation (*n* = 32). (**D**) Scale plot illustrating the lw, rc, smc, sr and vip for each of the eCBome predictors in the variable-response prediction model. (**E**) PLSR correlation plot between predicted and measured LBP according to the selected eCBs mediator subset, construct on all observations without cross-validation (*n* = 32). (**F**) Scale plot illustrating the lw, rc, smc, sr, and vip for each of the eCBs predictors in the variable-response prediction model. (**A**,**C**,**E**) The black line illustrated a perfect correlation (*R*^2^ = 1), the red line showed the measured correlation (five components for NEFA, four components for leptin, three components for LBP). (**D**,**F**) Color legend: red = smc (significance multivariate correlation); dark green = vip (variable importance in projections); light green = sr (selectivity ratio); dark blue = lw (loading weight); light blue = rc (regression coefficient). Abbreviations: see Table 1.

The cross-validated regression model constructed on all observations with four components had an *R*^2^ of 0.74, an adjusted *R*^2^ for the number of predictors of 0.60, and a RMSEP of 0.49. For our predictive approach, we performed leave-on-out cross validation on our model built on 80% of our sample, called the training set. Cross-validation is an important step to avoid overfitting the model if the finality is to build one that would have performance in predicting specific variable-response according to chosen predictors from new samples, called the test data (20% remaining observations). The model built of the training set was characterized by an *R*^2^ of 0.79, an RMSEP of 0.398, and an adjusted *R*^2^ of 0.63 (Figure 3A). Once the model is built, it is important to test if applying this model on the new samples, independent from the training set, allow to predict leptin in those samples. As we actually know the concentration of leptin of these samples, because they are the remaining 20% of the data set, we can estimate whether the original model has a good predictive performance. When the model was applied to the test data the *R*^2^ is 0.89 (Figure 3B), which is close from the train data *R*^2^ and the RMSEP was 0.75, which is acceptable given the number of observations used to construct the model. This result suggests that our model is performant enough to predict with approximate accuracy the NEFA from new samples according to the select eCBome profile. Once the model was validated, we asked our self which are predictors influencing the most the model. These analyses were performed using all the observations (*n* = 32), in order to take advantage of all of our biological resources, but still of the reduced ECB-subset. The jack test performed on the cross-validated model including all the observations showed that the most influential eCB-related entities were DHEA, AEA, DPEA, OEA, and SEA, among these mediators, AEA had the largest positive influence on NEFA and DPEA the largest negative influence on NEFA (Table 2).

#### 3.3.2. Leptin Response Modeling

The best performance was obtained with PLSR, for which the first three components captured 93% of the variation in the predictors and 40% of leptin variation. The non-cross-validated model had an *R*^2^ of 0.42, an adjusted *R*^2^ of 0.18 and RMSEP of 0.75 (Figure 3C). Cross-validated analysis on 80% of the observations failed to construct a model performant enough to predict leptin levels in new samples originating from the same population and according to the profile of eCBome mediators selected (Figure S1A,B).

Nevertheless, the construction of a linear model on all observations in a descriptive approach allows for the isolation of some relevant eCBome mediators for our specific cohort: EPA, DPA, EPEA, and DHEA were important drivers of leptin response (Figure 3D). Subsequent whole cross-validated modeling with a reduced number of predictors, selected on the basis of the rCCA-output, showed that the coefficient of regression for both DHEA and EPEA was significantly different from 0, following jack test (Figure S1C), suggesting their relevance in predicting leptin response in our cohort.

#### 3.3.3. LBP Response Modeling

The best performance was obtained with PLSR, for which the first three components explained 88% of the variance in the eCBome mediators while explaining 59% of LBP variance. When we built up a cross-validated model with PLSR using 1 component to predict the LBP response according to the eCBome mediators selected from the exploratory analysis, the *R*^2^ of the model was 0.25, the adjusted *R*^2^ 0.03, which is equivalent to 0, and the error was 0.85 which is high (Figure S2). This regression model was not performant enough to allow prediction of LBP from new observations, and therefore, was unable to generalize the variable response for the other cohort. Although we could not construct a predictive model, we seek to isolate the eCBs that influence the most the LBP response within our cohort. For this, the 32 observations were used to extract as much as information. The regression model built in a descriptive approach without performing cross-validation had a prediction error of 0.63, a *R*^2^ of 0.59 and an adjusted *R*^2^ of 0.47 (Figure 3E), meaning that we could estimate the value of LBP from our specific cohort according to the value of the selected eCBs. In this non cross-validated model, the most relevant eCBs variables according to the filter methods used for variable selection were DPEA, AEA, and EPEA in our cohort (Figure 3F).

### 3.4. Univariate Analysis on Quartiles

**Figure 4:**
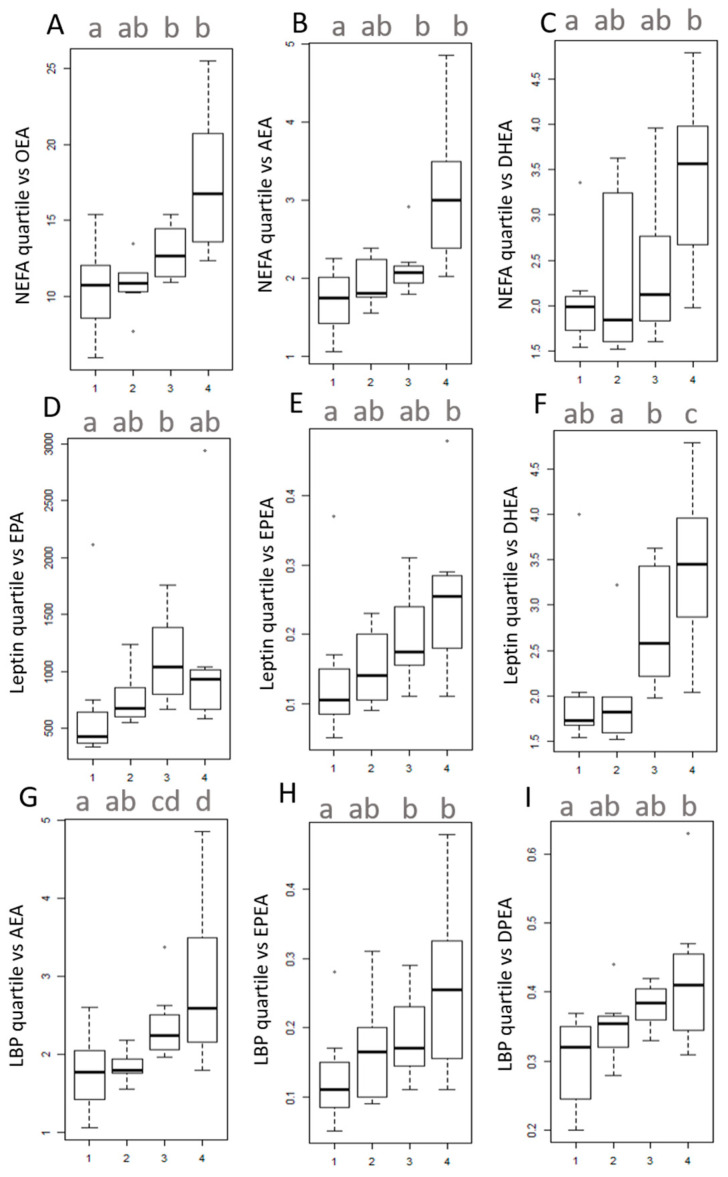
Univariate tests on quartiles. The number 1 corresponds to the lower quartile, while the number 4 corresponds to the upper quartile. (**A**,**B**) Boxplots of the concentration of (**A**) OEA, (**B**) AEA, (**C**) DHEA, according to NEFA quartiles. (**D**–**F**) Boxplots of the concentration of (**D**) EPA, (**E**) EPEA, (**F**) DHEA, according to leptin quartiles. (**G**–**I**) Boxplots of the concentration of (**G**) AEA, (**H**) EPEA, (**I**) DPEA, according to LBP quartiles. Data with different superscript letters are significantly different at *p* < 0.05, according to the post-hoc ANOVA statistical analysis (**C**,**F**), or Kruskal-Wallis multiple comparisons test (**A**,**B**,**D**,**E**,**G**–**I**). Abbreviations: see Table 1.

In three independent analyses, our entire data set was split into four groups according to the quartiles of the distribution of the metabolic variable of interest, thus converting the continuous variables into categorical variables using quartiles. We then measured the distribution of the relevant eCBome mediators, revealed upon multivariate analyses, in each of those new groups, and assessed whether the groups were statistically different from each other using univariate statistical tests. When observations were split according to NEFA quartiles, ANOVA analysis showed that the fourth quartile group had significantly higher DHEA levels compared to quartile one (Figure 4C). Kruskal–Wallis analysis showed that the fourth quartile group had significantly higher AEA and OEA levels compared to quartiles 1 and 2 (Figure 4A,B). No significant results were found for DPEA and SEA (data not shown). When observations were split according to leptin quartiles, individuals in the upper quartiles had significantly higher levels of DHEA and EPEA, when compared to those in the lower quartiles (Figure 4E,F). Comparison of the groups for EPA following Kruskal–Wallis test showed that the third quartile group had significantly higher EPA levels compared to quartile 1 (Figure 4D). No significant difference in levels were found for DPEA (data not shown). When observations were split according to BP quartiles, individuals in the upper quartiles had significantly higher levels of EPEA, AEA, and DPEA (Figure 4G–I).

## 4. Discussion

Obesity and ensuing metabolic disorders are becoming epidemic. In the healthy state, as well as in any pathological condition, viewing all biological entities as part of an entire biological system is crucial to better understanding the complexity of organisms and the etiology of a disease. In this context, the eCBome is a relevant system to study. The eCBome is a complex lipid-signaling system composed of tens of bioactive lipid mediators related to eCBs, their membranes and nuclear receptors, as well as anabolic and catabolic enzymes, and includes the eCB system [8].

Increasing evidence suggests that physiological functions such as energy balance, appetite, and glucose/lipid metabolism are partially under the regulation of the eCBome [7,12,31,32]. Accordingly, the contribution of a dysregulation in the eCBome was highlighted in a number of pathological conditions and related complications, such as type 2 diabetes, fatty liver disease, obesity, and certain neurological disorders [4,8,33,34]. The study of this system is, therefore, necessary to better understand the mechanisms underlying such metabolic disorders.

The amount of biological data produced by high-throughput technologies across the whole spectrum of biology is substantial. In parallel, dimension reduction methods are promising approaches for data integration [19]. While offering both novel opportunities and challenges to unravel multifactorial diseases such as obesity, and identify new therapeutic perspectives, these techniques also raise questions regarding how to use them to draw meaningful biological conclusions.

Initially, we had a large volume of data, both in terms of the eCBome, and in terms of metabolic parameters of heterogeneous nature. Most of the lipids associated with the eCBome have been characterized, but only a limited number of studies have documented or explored this system in a comprehensive picture at a system biology level in the context of obesity. Accordingly, our study aimed at identifying new specific dialogue features in the cross-talk between eCB congeners and metabolic parameters in obese or overweight individuals with prediabetes and metabolic syndrome. Seeking for such relationships naturally led us to consider the correlation matrix in our analytical approach. However, being a bivariate method, the matrix only analyzes one pair of variables at a time, ignoring the others. Moreover, reading the matrices becomes complicated when a large number of variables is involved. Unlike univariate analysis, multivariate analysis brings together a set of statistical methods that take into account several data variables at the same time. Thus, these statistical methods can examine more complex interplays and find new data patterns more accurately representative of biological systems [35].

In order to reconcile, at the same time, the need to isolate relevant variables, and to take into account all the data as part of a whole, and thus, examine the interactions existing between them, we performed a preliminary rCCA. As a reminder, CCA is an integrative analysis is an unsupervised method seeking for the largest correlation existing between a linear combination of the variables in the first set and a linear combination of the variables in the second set [27]. In this way, rCCA is an interesting extension to the correlation matrix and can provide additional insights that would not be obtained by performing univariate analysis, or by analyzing each data set alone [27]. The graphical examination of the relationships through both rCCA-derived circle plot and network showed that specific subsets of the eCBome were associated with several metabolic parameters, including leptin, NEFA, LBP, and FM. Importantly, these four variables, although of heterogeneous nature, are known to be altered upwards in obesity. It is worth noting that results for FM are consistent with previous studies showing that fat distribution is an important determinant of peripheral eCBome mediator levels [11,36,37,38].

Importantly, following selection of the relevant variables, the subsequent Spearman’s correlation matrix further corroborated the existence of a general correlation between leptin, NEFA, and LBP and several eCB congeners. Thus, our analyses not only highlighted the importance of using a preliminary exploratory analysis to reduce the dataset, but also illustrated the added value of combining both multivariate and univariate approaches. Finally, both univariate and multivariate approaches demonstrate collinearity between NAE and between MAGs, consistent with the fact that within each eCB related-family, mediators share biosynthetic and catabolic pathways and enzymes [5].

By definition, the rCCA is explorative, meaning that the above analyses opened doors for more in-depth investigations but did not statistically assess whether the correlations between the biological variables and eCB congeners are significant. Thus, to confirm our assumptions we performed a PCA on plasma eCBome-related mediators. In this way, we extracted the first two linear relationships that best explained the variance across the quantified eCBome. Using the PCR principle, we correlated those components with the metabolic variables highlighted by rCCA. Our analyses showed positive associations between the first components and the variables leptin, NEFA, LBP, and FM.

Next, we modeled and validated variable responses through multiple linear regression. For this, either PCR or PLSR were performed and predictors selected on the basis of the network generated in the rCCA. We performed cross-validation when we wanted to go beyond descriptive analyses. From a methodological point of view, our results aligned with the literature, showing that PLSR often outperformed PCR in terms of predictive power, notably due to its supervised nature [39,40]. Finally, our analyses also highlighted the importance of cross-validating the model to avoid overfitting in a predictive approach.

Regarding NEFA, the prediction model built on 80% of the observations by PLSR was performant enough to predict with approximate accuracy the NEFA levels from the remaining samples according to the selected eCBome profile. Although very interesting, our validation is limited by its internal characteristic. Thus, we should ideally confirm these results upon external validation. By analyzing the importance of each predictor for the explanation of NEFA-responses, we isolated SEA, OEA, AEA, DPEA, and DHEA as important predictors. Among them, AEA had the largest positive influence, while DPEA had the largest negative influence on NEFA levels.

From a biological point of view, NEFA, are circulating protein-bound lipids and represent an important energy fuel, some of them also having cellular signaling function [41]. They either originate from intestinal absorption of dietary fats or from the adipose tissue through lipolysis. Importantly, lipolysis is accentuated upon insulin resistance which results in higher levels of circulating NEFAs, which can subsequently reach target organs such as the liver or skeletal muscle, increasing NEFA fluxes to these organs [42]. High NEFA levels are a marker of altered lipid metabolism and represent a common feature of obesity-related conditions [43]. PCA analysis showed that individuals with higher NEFA levels had higher eCBome tone. In a more targeted approach, analysis of quartiles showed that these individuals had significantly higher levels of OEA, AEA, and DHEA. Interestingly, OEA was previously shown to exert pro-lipolytic effects both in vivo and in vitro, notably via the activation of PPAR α [44,45,46]. In view of our results and given that no data in the literature yet exist to invalidate our assumption, we postulate that DHEA might exert pro-lipolytic effects such as OEA. Finally, our data regarding the CB_1_-ligand AEA, might appear as unexpected as the literature shows that it has pro-lipogenic properties in the adipose tissue and in the liver, promoting fat accumulation, notably through the activation of CB_1_ and PPARɣ [47,48,49]. However, a previous study demonstrated a clear correlation between plasma free fatty acids and AEA levels regardless of the fasting state in healthy women [50]. Further studies are needed to find out whether or not this relationship, specific of the blood compartment, is a direct reflection of AEA concentrations within metabolic tissues. Furthermore, we cannot exclude that the direction of the relationships might vary alongside disease progression.

Regarding leptin, at completion of PCR and PLSR analyses, we concluded that our prediction model had a limited performance to predict leptin using a reduced eCBome profile. In more details, after several adjustments, it appeared essential to reduce the number of predictors, and the final model only included the eCBome members AEA, OEA, DHEA, and EPEA, which were notably revealed by the rCCA. The unsupervised PCR approaches showed that these four variables alone explained 37% of the variations in leptin values in our cohort. Nevertheless, under cross-validation, the performance of the model decreased to 20%, and 10% when *R*^2^ was adjusted for the number of predictors.

However, the PCA carried out on the quantified eCBome showed a positive and significant correlation between leptin and the first principal components (i.e., the one summarizing as much as possible the variance in our eCBome). Furthermore, if a color code, corresponding to the levels of leptin, was applied to each observation, the variable plot showed a gradation of leptin following that of dimension one. As the coefficient of the first component was positive, we can conclude that a stronger eCBome tone would be associated with higher leptin levels. Finally, the PCR analyses, the rCCA, and the correlation matrix all converged to indicate a strong correlation between leptin and both DHEA and EPEA. These eCB congeners were further investigated by quartiles analyses. Our data confirmed that individuals in the upper quartiles had significantly higher levels of DHEA and EPEA, when compared to those in the lower quartiles. This result was unprecedented, and yet not completely surprising in view of the fact that high leptin levels are a biomarker of obesity, and hence systemic inflammation, which can also be exacerbated by excessive leptin (reviewed in [51]). By contrast, EPEA and, particularly, DHEA are known to be anti-inflammatory mediators, and their levels may be increased as an adaptive response to this condition [52,53,54]. Under more physiological conditions, leptin is a satiety hormone, controlling food intake centrally [55]. Previous studies have demonstrated in mice that this hormone negatively regulates the hypothalamic biosynthesis of orexigenic eCBs (i.e., AEA and 2-AG), thus further contributing to appetite inhibition [56,57]. Accordingly, reduced leptin sensitivity might subsequently lead to higher levels of AEA, which one recent report showed to be positively correlated to other NAEs, including EPEA and DHEA, using PCA in a heterogeneous population [11]. However, we did not have a healthy control group to confirm the occurrence of leptin resistance in our obese patients. In summary, further studies are needed to confirm our assumptions on the cross-talk between eCB congeners and leptin.

Regarding our analysis of the LBP response, the cross-validated analysis performed on all observations produced a predictive model with an *R*^2^ close to 0. Accordingly, we were unable to explore the relationship in a predictive approach. As such, we failed to construct a model performant enough to predict LBP levels in new samples originating from the same population according to the eCBome profile selected. Nevertheless, the construction of a linear model including all observations in the framework of a descriptive approach allowed us to isolate some relevant eCBome mediators for our specific cohort—i.e., DPEA, EPEA, and AEA—as important drivers of LBP levels in our specific cohort.

From a biological point of view, LBP is a surrogate marker of endotoxin translocation and, thus, is viewed as a marker of chronic systemic inflammation. Accordingly, its serum concentration have been shown to be strongly correlated with obesity-associated metabolic disorders [58,59,60,61]. Interestingly, the mediators found to be significantly higher in individuals from the upper LBP quartiles (i.e., DPEA, EPEA, and to some extent, AEA), have been reported to exert anti-inflammatory effects [52,62,63,64]. Therefore, this finding corroborates our above hypothesis that such mediators are elevated in the blood as an adaptive response aiming at counteracting inflammation. However, CB1 activation by eCBs such as AEA can also exacerbate inflammation [65,66].

In terms of perspective, it would be interesting to apply similar methodological approaches in other cohorts to explore the cross-talks between eCB-related bioactive lipids and metabolic parameters in other metabolic diseases such as type 2 diabetes or non-alcoholic fatty liver disease. Above all, it would be important to compare our results with those in a healthy population to verify if the relationships highlighted in this study are specific for the characteristics of our cohort. Finally, a recent report confirmed that some gut bacteria are associated with variation in eCB congeners, independently from adiposity measures [11]. Thus, it would be interesting to see if probiotic treatments of individuals with the metabolic syndrome strengthens the correlations observed here. Finally, given the relatively small number of subjects within the analyzed cohort, we acknowledge that replicating our findings on larger cohorts will be helpful to further extend and validate the novel methods proposed in this study.

In conclusion, the unsupervised methods used in this study (rCCA, PCA) highlighted/revealed the existence of an association between the eCBome and specific metabolic biological parameters (i.e., Leptin, NEFA, and LBP), while multiple linear regression using PCR and ¨PLSR extended our findings to identify relevant eCBome mediators that might greatly influence metabolic variable response. Interestingly, the selected metabolic parameters are part of biological pathways that are markedly altered in the context of the metabolic syndrome. Beyond its primary objective of describing a dataset and finding new potential links, this paper experimented a methodological approach based on the conjunction of several multivariate approaches. Our manuscript thus highlights the value of combining these different approaches to univariate methods in order to unravel specific cross-talks between two large datasets. Our methodological study finds its main limitation in the number of subjects, which may have limited the construction of a predictive model. Nevertheless, our data ultimately support the importance of cross-validation to avoid overfitting and, thus, generalizing models, whose validation is specific to a given cohort. Pending confirmation from larger studies, our results find their interest in opening new avenues to further exploring how the eCBome affects the organism in the context of obesity.

## Figures and Tables

**Table 1:** Endocannabinoids and metabolic parameters abbreviations.

Endocannabinoids		Metabolic Parameters	
AEA	*N*-arachidonoyl-ethanolamine	FM	fat mass
2-AG (AG12)	1(3)- and 2-arachidonoyl-glycerol	HbA1c	glycated hemoglobin A1c
DHEA	*N*-docosahexanoyl-ethanolamine	LBP	lipopolysaccharide-binding protein
DHA	docosahexaenoic acid	LDL	low density lipoprotein
2-DHG (DHA12)	1(3)- and 2-docosahexaenoyl-glycerol	NEFA	non-esterified fatty acids
2-DPG (DPAG12)	1(3)- and 2-docospentaenoyl-glycerol(n-3)	TC	total cholesterol
DPEA	*N*-docosapentaenoyl-ethanolamine(n-3)	T.H	total/HDL cholesterol
DPA	docosapentaenoic acid (n-3)		
EPA	eicosapentaenoic acid		
EPEA	*N*-eicosapentaenoyl-ethanolamine		
LEA	*N*-linoleoyl-ethanolamine		
2-LG (LG12)	1(3)- and 2-linoleoyl-glycerol		
OEA	*N*-oleoyl-ethanolamine		
2-OG (OG12)	1(3)- and 2-oleoylglycerol		
PEA	*N*-palmitoyl-ethanolamine		
SEA	*N*-stearoyl-ethanolamine		

**Table 2:** Jack test results table showing the PLS regression coefficients using four components for the cross-validated NEFA prediction model constructed from all observations (*n* = 32) and using the reduced ECB-subset as predictors.

Predictors	Estimate	Std Error	T Value	Pr(>|t|)	*
PEA	0.049287	0.203249	0.2425	0.809993	
SEA	−0.575454	0.274860	−2.0936	0.044567	*
OEA	0.597778	0.267343	2.2360	0.032689	*
LEA	−0.139934	0.267431	−0.5233	0.604519	
AEA	0.898707	0.201773	4.4541	0.000102	***
EPEA	0.494766	0.264972	1.8672	0.071350	
DPEA	−0.625028	0.221271	−2.8247	0.008202	**
DHEA	−0.371274	0.177796	−2.1687	0.037895	*
DPA	−0.020114	0.206108	−0.0976	0.922886	
EPA	0.271866	0.217275	1.2513	0.220202	
DHA	0.200733	0.182672	1.0989	0.280285	

Abbreviations: see Table 1. * *p* < 0.01; ** *p* < 0.001; *** *p* < 0.000.

## Data Availability

The data presented in this study are available on request from the corresponding author.

## Supplementary Materials

The following are available online at https://www.mdpi.com/2073-4409/10/1/71/s1. Figure S1: Attempt to construct a variable-response predictive model for leptin according to ECBs subset, Figure S2: Attempt to construct a variable-response cross-validated model for LBP according to ECBs subset.
