# Supplementary material for: Linking the Endocannabinoidome with Specific Metabolic Parameters in an Overweight and Insulin-Resistant Population: From Multivariate Exploratory Analysis to Univariate Analysis and Construction of Predictive Models
Source: Cells. 2021 Jan 5;10(1):71. doi: 10.3390/cells10010071 (PMC7824762; doi:10.3390/cells10010071)
Supplement: Supplementary file 1 [file cells-10-00071-s001.pdf]

Supplementary Materials: The following are available online at www.mdpi.com/2073-4409/10/1/71/s1,

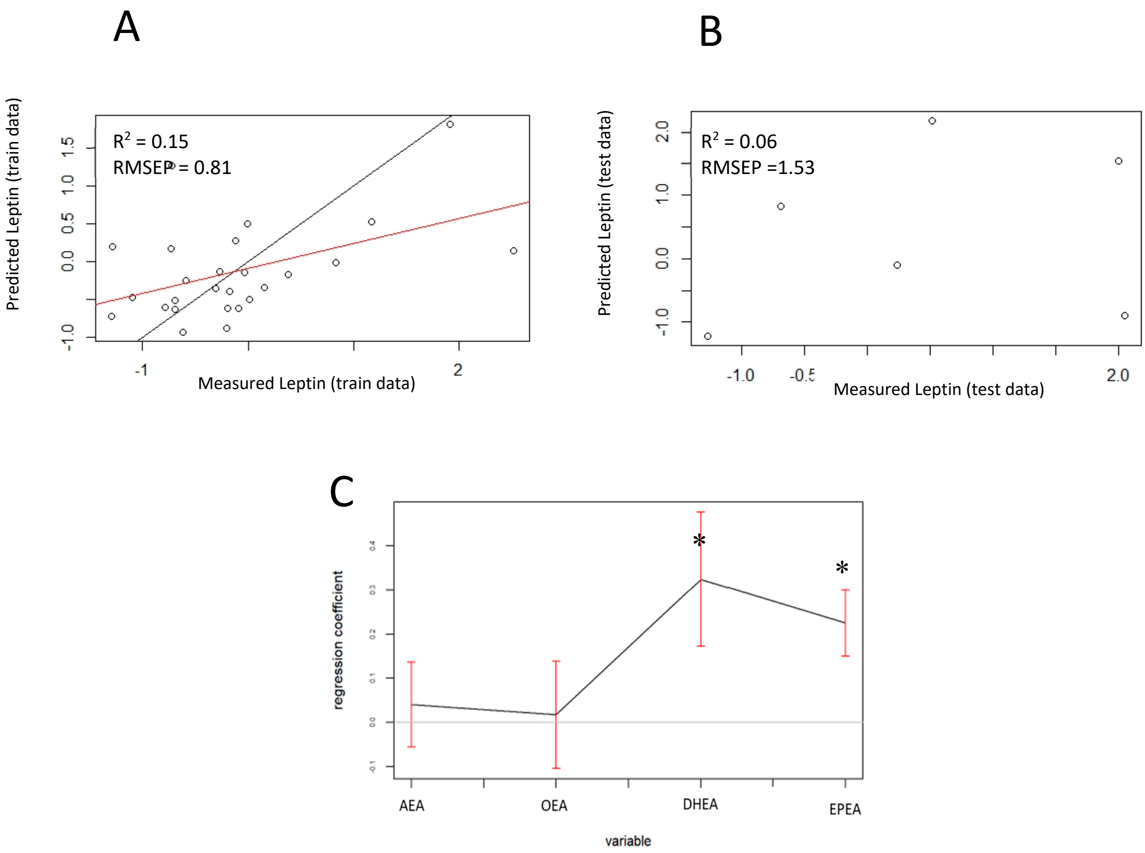

Figure S1: Attempt to construct a variable-response predictive model for leptin according to ECBs subset, and validation of the model on a test data set. **(A-B)** PCR correlation plot between predicted and measured leptin levels according to the selected ECBs subset using 4 components, illustrating the predictive quality of leptin in the **(A)** training group (n=26) and **(B)** the test set (n=6) (2 components). This last model was constructed using PCR which showed better performance than PLSR. This is justified by the fact that, while capturing the same amount of information, PCR had a reduced RMSEP error. The black line illustrated a perfect correlation (R^2=1), the red line showed the measured correlation. **(C)** Jack test results table showing the PCR regression coefficients using 2 components for the cross-validated leptin prediction model constructed from all observations (n=32).

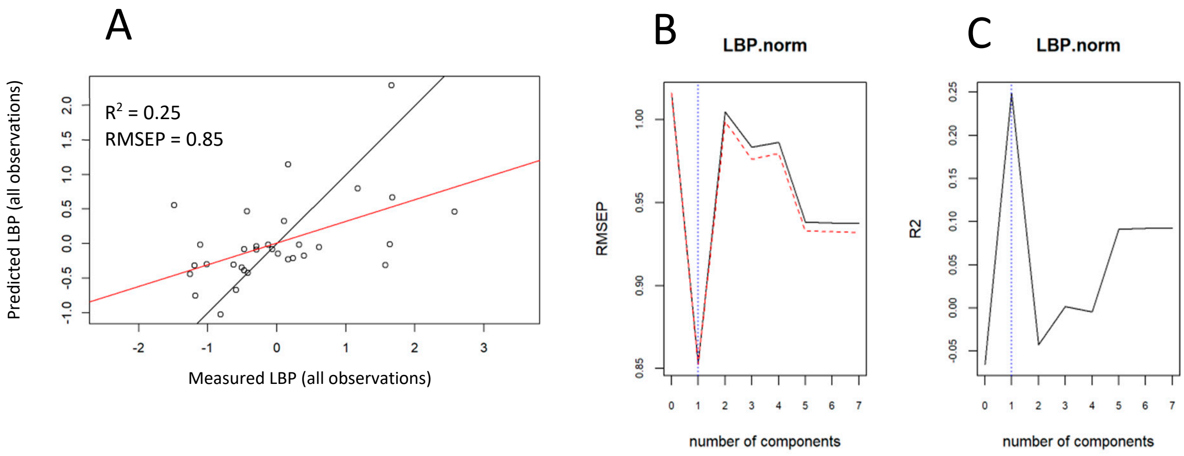

Figure S2: Attempt to construct a variable-response cross-validated model for LBP according to ECBs subset, and validation of the model on all observations (n=32). **(A)** PLSR correlation plot between predicted and measured LBP levels according to the selected ECBs subset, illustrating the predictive quality of LBP using 1 component. The black line illustrated a perfect correlation (R^2=1), the red line showed the measured correlation. **(B-C)** Validation showing the RSMEP **(B)** and R^2 **(C)** according to the number of components included in the model.
